# Billroth II With Braun Anastomosis Versus Roux‐En‐Y Reconstruction Following Distal Gastrectomy: A Systematic Review and Meta‐Analysis

**DOI:** 10.1002/wjs.70256

**Published:** 2026-02-10

**Authors:** Wendy Chang, Lucas Monteiro Delgado, Jessica Ng, Bryan Tran

**Affiliations:** ^1^ Department of Surgery Gosford Hospital Gosford New South Wales Australia; ^2^ University of Queensland Herston Queensland Australia; ^3^ Department of Surgery Federal University of Minas Gerais Belo Horizonte Minas Gerais Brazil; ^4^ Gold Coast University Hospital Gold Coast Queensland Australia

## Abstract

**Introduction:**

The efficacy of Billroth II with Braun (BIIB) anastomosis in laparoscopic distal gastrectomy remains uncertain. We aimed to perform a systematic review and meta‐analysis comparing BIIB with Roux en Y (RY) for distal laparoscopic gastrectomy.

**Methods:**

We systematically searched PubMed, Embase, and Cochrane for studies comparing BIIB versus RY in adult patients undergoing distal gastrectomy. We computed risk ratios (RRs) for binary outcomes and mean differences (MDs) for continuous outcomes, with 95% confidence intervals (CIs). Heterogeneity was assessed with *I* ^2^ statistics. Statistical analyses were performed using R software, version 4.2.3.

**Results:**

We included 10 studies, comprising a total of 1377 patients. BIIB was associated with a lower anastomotic time (MD 7.82 min; 95% CI −11.99 to −3.65; *p* = 0.0002; *I* ^2^ = 99%), intraoperative blood loss (MD −17.88 mL; 95% CI −31.00 to −4.76; *p* = 0.0076; *I* ^2^ = 91%), and operative time (MD −21.67 min; 95% CI −28.62 to −14.72; *p* < 0.01; *I* ^2^ = 80%). Also, BIIB group was associated with a higher incidence of bile reflux when compared to the RY group (RR 3.10; 95% CI 1.75 to 5.50; *p* < 0.0001; *I* ^2^ = 74%). There were no significant differences between BIIB and RY for anastomotic leakage rate, number of retrieved lymph nodes, gastritis, residual food, time to first exhaust, length of hospital stay, time to liquid diet, and adverse events.

**Conclusion:**

In adult patients undergoing distal gastrectomy, BIIB was associated with a shorter operative, anastomotic time, and intraoperative blood loss, with an increased incidence of bile reflux. BIIB may be an easier and feasible alternative to RY, especially in patients who should avoid excessive exposure to anesthesia.

## Graphical abstract

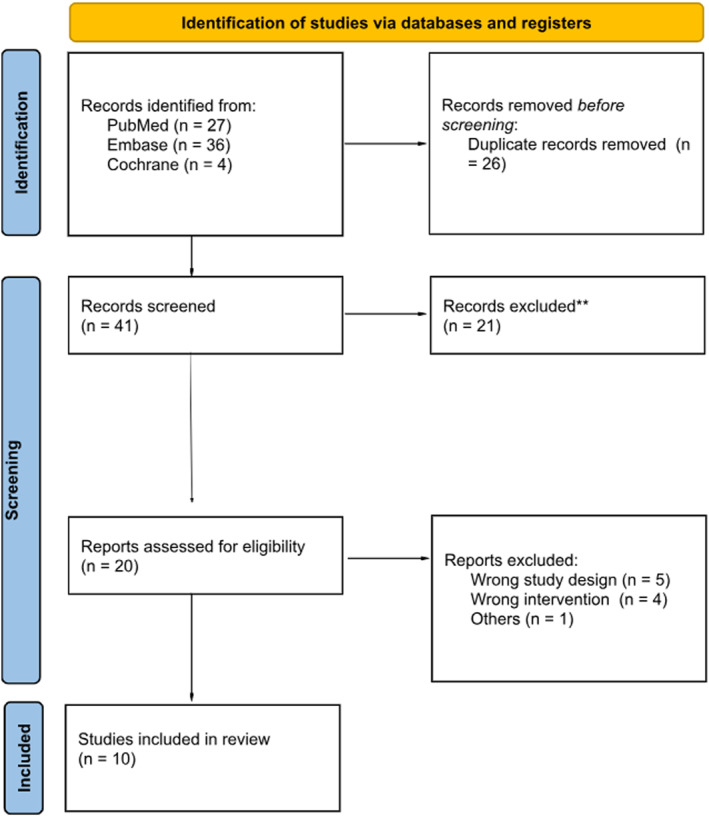

## 1 Introduction

Gastric cancer remains a major global health problem, being the fifth leading cause of new cancer diagnoses worldwide [1]. Distal gastric cancer often requires distal gastrectomy with resection of the pylorus with reconstruction of their alimentary tract [2]. There are several reconstruction techniques including: Billroth I (BI), Billroth II (BII), Billroth II with Braun anastomosis (BIIB), conventional Roux‐En‐Y (RY), and uncut RY. BII and RY remain the most frequently performed reconstruction techniques, serving as surgical standards for decades [3, 4]. However, BII procedures are associated with bile reflux gastritis due to resection of the pylorus and its sphincter, which acts as a valve blocking the flow of bile from the small bowel back into the stomach [2]. BII is also associated with afferent loop syndrome, gastric remnant carcinomas in animal models due to excess bile reflux, and ulcers [2].

To address these limitations, BIIB has been proposed as an alternative technique. Billroth first performed the BII procedure in 1885, in which both the gastric and duodenal stumps were closed and gastrojejunostomy anastomosis was performed [5]. Later, in 1892, Heinrich Braun introduced the Braun anastomosis, where a side‐to‐side anastomosis was created between the afferent and efferent jejunum [5]. By adding a side‐to‐side enteroenterostomy, this method aims to divert the bile flux distally into the efferent limb and into the small bowel, thereby reducing the incidence of bile reflux, alkaline reflux esophagitis, afferent loop syndrome, and associated complications [2, 5] Technically, it is a less complex surgery than RY, decreases the risk of internal hernia, Roux stasis syndrome, and is less likely to develop nutritional deficiencies [2, 6].

A meta‐analysis was performed comparing BIIB anastomosis and uncut RY for patients undergoing distal gastrectomy, and showed that BIIB was associated with lower operative time, less intraoperative blood loss, less postoperative complications, and reduced gastric emptying [7]. However, no comprehensive systematic review and meta‐analysis has been performed comparing BIIB anastomosis and conventional RY for distal gastrectomy. Therefore, we aimed to perform a systematic review and meta‐analysis in this patient population to assess the efficacy and safety of BIIB when compared to RY.

## 2 Methods

This systematic review and meta‐analysis was conducted following Cochrane recommendations and Preferred Reporting Items for Systematic Review and Meta‐Analyses (PRISMA) guidelines [8]. The protocol for this study was prospectively registered in the International Prospective Register of Systematic Reviews (PROSPERO) database under protocol number CRD420251062592.

### 2.1 Eligibility Criteria

Studies were considered eligible for inclusion when meeting all of the following criteria: (1) Adults patients (> 18 years); (2) undergoing laparoscopic distal gastrectomy (3) comparing BIIB versus RY; and (4) reporting at least one outcome of interest. Exclusion criteria included studies (1) involving children; (2) undergoing open surgery; or (3) not published as original articles.

### 2.2 Search Strategy and Data Extraction

We systematically searched Pubmed, Embase, and Cochrane databases from inception to June 2025, with the following search terms: “gastric cancer,” “gastrectomy,” “Billroth‐II with Braun” “B‐2‐B.” The complete search strategy is presented in the Supporting Information S1: Table S1. References from all included studies, previous systematic reviews and meta‐analyses were also manually searched to identify any additional studies. Data were independently extracted by two authors (W.C. and L.M.), following predefined search criteria. A template was developed for data extraction of relevant items, including study details (author, year of publication, study design, sample size, and time of follow‐up), participants (age and BMI), intervention, control, and outcome measure.

### 2.3 Endpoints and Subgroup Analysis

Our endpoints included anastomotic time, operative time, anastomotic leakage rate, number of retrieved lymph nodes, anastomotic bleeding, Clavien Dindo (CD) I–II, CDIII–IV, dumping syndrome, ileus, intraabdominal bleeding, blood loss, grade of bile reflux, grade of gastritis, intraoperative blood loss, length of hospital stay, time to first exhaust, and time to liquid diet.

CD I–II was defined as complications requiring pharmacological treatment with drugs other than those allowed for grade I complications (e.g., antibiotics, blood transfusion, total parenteral nutrition). CD III–IV was defined as requiring surgical complications requiring surgical, endoscopic, or radiological intervention (grade III), or life‐threatening complications requiring ICU management (grade IV) [9].

### 2.4 Risk of Bias Assessment

RCTs were appraised with the Cochrane Collaboration's tool for assessing risk of bias in randomized trials (RoB‐2), with 5 domains: selection, performance, detection, attrition, and reporting [10]. Risk Of Bias in Non‐randomized Studies of Interventions (ROBINS‐I) tool was used to evaluate the cohort studies, with 7 domains: confounding, selection of participants, classification of interventions, deviations from intended interventions, missing data, measurement of outcomes, and reported results [11]. Authors (W.C. and L.M.) independently assessed the risk of bias. Disagreements were resolved with a third author (B.T.).

Publication bias was assessed by visual analysis of the funnel plot to evaluate symmetric distribution of trials with similar weight [12]. No quantitative assessment of small studies or publication was performed due to the small number of studies included in each individual outcome.

### 2.5 Sensitivity Analyses

We performed leave‐one‐out sensitivity analyses for anastomotic time to assess the effects of influential studies on the pooled analysis. Studies were sequentially removed and the data were reanalyzed to ensure the stability of the pooled effects.

### 2.6 Statistical Analysis

We pooled risk ratios (RR) and mean differences (MD) with 95% confidence intervals (CI) for binary and continuous outcomes, respectively. DerSimonian and Laird random‐effects models were employed for all endpoints due to the heterogeneity in methodology and demographics across the individual studies. We assessed heterogeneity with *I* ^2^ statistics and Cochran *Q* test; *p* values < 0.10 and *I* ^2^ > 40% were considered significant for heterogeneity. All statistical analyses were performed using R software version 4.3.2 (R foundation, Vienna, Austria).

## 3 Results

### 3.1 Study Selection and Characteristics

**FIGURE 1:**
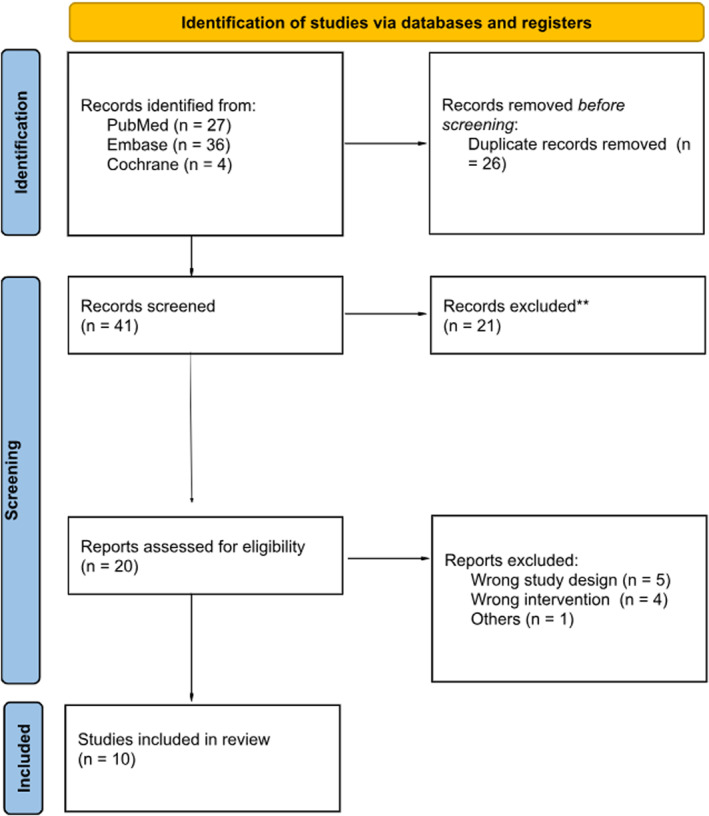
PRISMA flow diagram of study selection.

Our initial search found 67 articles in June 2025. After removing duplicate reports and applying the eligibility criteria, 20 studies were selected for full‐text review, as detailed in Figure 1. Of these, 10 studies were included in this systematic review and meta‐analysis [13, 14, 15, 16, 17, 18, 19, 20, 21, 22]. The main reasons for exclusion were wrong study design and wrong intervention. A total of 1377 patients were included, of whom 831 (60%) underwent BIIB surgery. Mean patients’ ages ranged from 54.1 to 67.3 years. The baseline characteristics were mostly compared between groups, as shown in Table 1.

**TABLE 1:** Design and characteristics of the studies included in the meta analysis.

First author, year	Country	Design	No. of patients		Female, %		Mean age, years		Mean BMI		ASA I, %		ASA II, %		ASA III, %		ASA IV, %	
			BIIB	RY	BIIB	RY	BIIB	RY	BIIB	RY	BIIB	RY	BIIB	RY	BIIB	RY	BIIB	RY
Chen et al. [23]	China, single‐center	R‐Obs	94	57	28.7	36.8	60.29	61.72	21.53	22.25	86^a^	85.1^a^	86^a^	85.1^a^	14	14.9	0	0
Chi et al. [13]	China, single‐center	R‐Obs	54	51	35.2	39.2	65.3	67.3	21.8	22.0	44.4	49	44.4	41.2	11.2	9.8	0	0
Choi et al. [18]	China, single‐center	P‐Obs	26	40	30.8	30	59.7	57.2	23.4	23.7	15.4	45	84.6	50	0	5	0	0
Cui et al. [15]	South Korea, single‐center	R‐Obs	26	30	42.3	26.7	60.1	57.6	23.3	24.0	50	60	50	40	0	0	0	0
Park et al. [21]	South Korea, multicenter	R‐Obs	183	67	35	37.3	60.0	60.4	23.7	23.7	57.9	56.7	37.2	37.3	4.9	6	0	0
Parthasarathy et al. [20]	India, single‐center	RCT	26	28	15.4	21.3	54.1	54.6	19.5	17.4	NA	NA	NA	NA	NA	NA	NA	NA
Shishegar et al. [16]	Iran, multicenter	RCT	42	42	38.09	26.19	59.8	57.3	22.7	23.9	57.1	64.3	42.9	35.7	0	0	0	0
Xiao et al. [19]	China, single‐center	R‐Obs	110	41	33.6	31.7	57.65	58.10	23.13	23.23	18.2	24.4	73.6	70.7	8.2	4.9	0	0
Yalikun et al. [14]	China, single‐center	R‐Obs	145	102	33.1	29.4	62	63	23.4	22.8	42.1	44.1	49.6	41.2	8.3	14.7	0	0
Zhong et al. [17]	China, single‐center	R‐Obs	125	88	26.4	30.7	NA	NA	NA	NA	47.2	39.8	43.2	51.1	9.6	9.1	0	0

*Note:* BMI categories: underweight (< 18.5 kg/m^2^), normal weight (18.5–23.9 kg/m^2^), overweight (24–27.9 kg/m^2^), obese (≥ 28 kg/m^2^). Distribution: BII: underweight 4.7%, normal 43.8%, overweight 43.8%, obese 7.8%. RY: underweight 6.6%, normal 52.7%, overweight 31.7%, obese 9%.

Abbreviations: ASA, American Society of Anesthesiologists Physical Status Classification; BIIB, Billroth‐II with Braun; BMI, Body Mass Index; P‐Obs, Prospective observational study; RCT, Randomized controlled trial; R‐Obs, Retrospective observational study; RY, Roux‐en‐Y reconstruction.

^a^ These values represent both ASA I and ASA II percentages.

### 3.2 Intraoperative Outcomes

**FIGURE 2:**
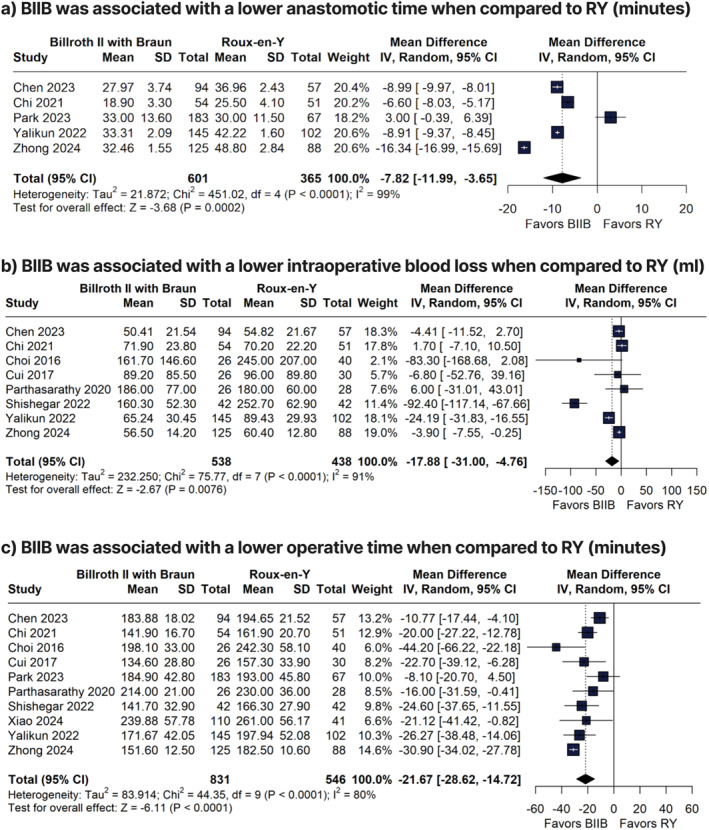
Forest plots of anastomotic time, intraoperative blood loss, and operative time comparing Billroth II with Braun and Roux‐en‐Y reconstruction.

**FIGURE 3:**
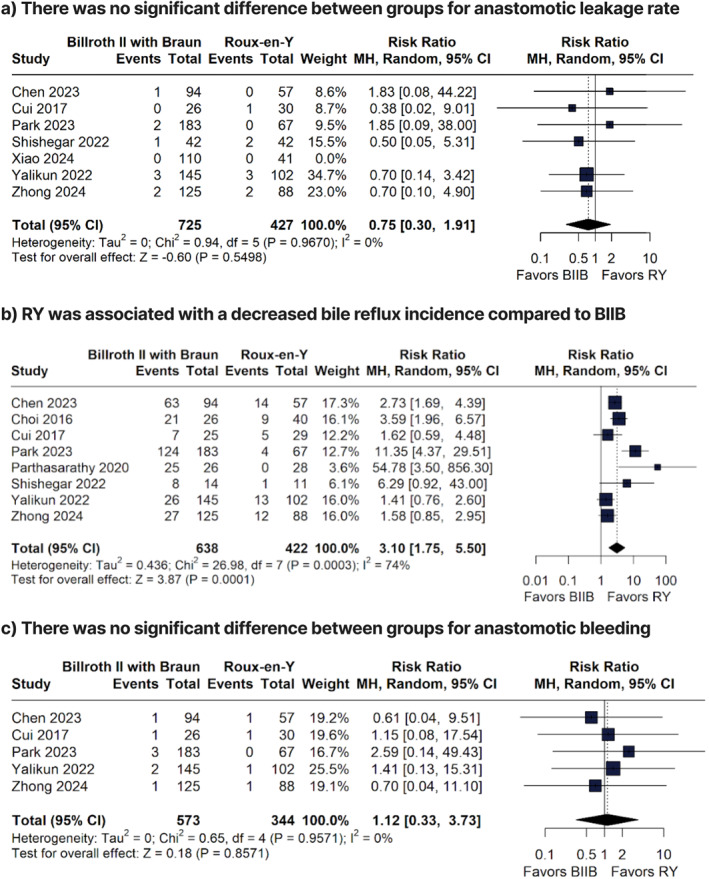
Forest plots of anastomotic leakage, bile reflux, and anastomotic bleeding comparing Billroth II with Braun and Roux‐en‐Y reconstruction.

When compared to RY, BIIB was associated with a lower anastomotic time (MD −7.82 min; 95% CI −11.99 to −3.65; *p* = 0.0002; *I* ^2^ = 99%; Figure 2a), lower intraoperative blood loss (MD −17.88 mL; 95% CI −31.00 to −4.76; *p* = 0.0076; *I* ^2^ = 91%; Figure 2b), and lower operative time (MD −21.67 min; 95% CI −28.62 to −14.72; *p* < 0.01; *I* ^2^ = 80%; Figure 2c). There were no significant differences between BIIB and RY groups for anastomotic leakage rate (RR 0.75; 95% CI 0.30 to 1.91; *p* = 0.5498; *I* ^2^ = 0%; Figure 3a) and number of retrieved lymph nodes (MD −0.12 nodes; 95% CI −2.78 to 3.02, *I* ^2^ = 96%; Supporting Information S1: Figure S1).

### 3.3 Adverse Events

**FIGURE 4:**
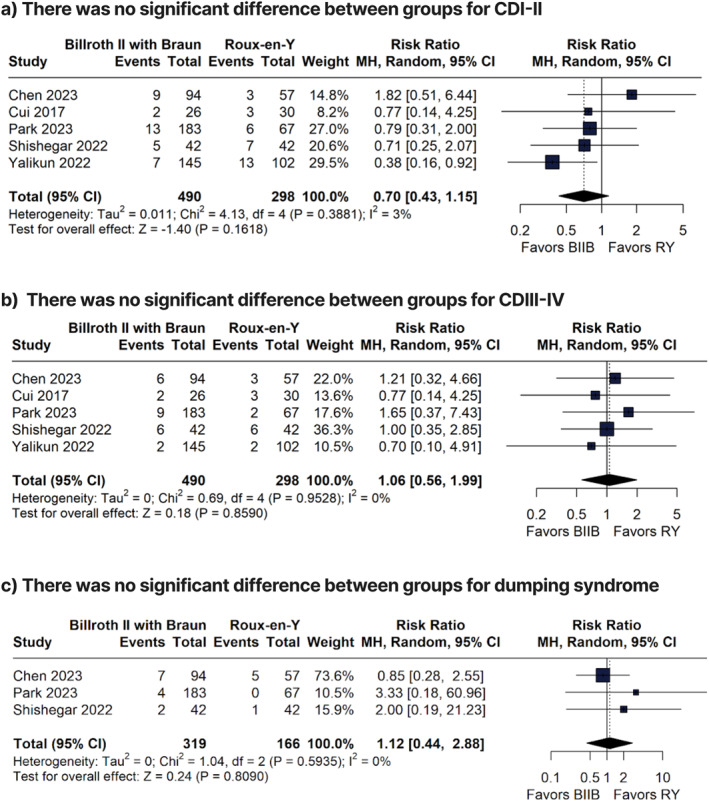
Forest plots comparing Clavien–Dindo grade I–II and III–IV complications and dumping syndrome between Billroth II with Braun and Roux‐en‐Y reconstruction.

There were no significant differences between groups for anastomotic bleeding (RR 1.12; 95% CI 0.33 to 3.73; *p* = 0.8571; Figure 3c), CDI–II (RR 0.70; 95% CI 0.43 to 1.15; *p* = 0.1618; *I* ^2^ = 3%; Figure 4a), CDIII–IV (RR 1.01; 95% CI 0.55 to 1.88; *p* = 0.9473; *I* ^2^ = 0%; Figure 4b), dumping syndrome (RR 1.06; 95% CI 0.56 to 1.99; *p* = 0.8590; *I* ^2^ = 0%; Figure 4c), ileus (RR 0.82, 95% CI 0.25 to 2.70, *p* = 0.7413, *I* ^2^ = 0%; Supporting Information S1: Figure S2), intraabdominal bleeding (RR 1.41; 95% CI 0.42 to 4.71; *p* = 0.5787; *I* ^2^ = 0%; Supporting Information S1: Figure S3), and pneumonia (RR = 0.76; 95% CI 0.26 to 2.19; *p* = 0.6044; *I* ^2^ = 0%; Supporting Information S1: Figure S4).

### 3.4 Endoscopic Outcomes

There were no significant differences between the two groups for Grade 1 (RR 1.23; 95% CI 0.99 to 1.53; *p* = 0.0663; *I* ^2^ = 68%; Supporting Information S1: Figure S5), grade 2 (RR 1.88; 95% CI 0.83 to 4.22; *p* = 0.1280; *I* ^2^ = 72%; Supporting Information S1: Figure S5), grade 3 (RR 1.23; 95% CI 0.23 to 6.56; *p* = 0.24; *I* ^2^ = 37%; Supporting Information S1: Figure S5) and grade 4 gastritis (RR 1.12; 95% CI 0.12 to 10.26; *p* = 0.8039; *I* ^2^ = 63%; Supporting Information S1: Figure S5).

There were no significant differences between the two groups for grade 1 (RR 0.98; 95% CI 0.66 to 1.45; *p* = 0.9058, I^2^ = 0%, Supporting Information S1: Figure S6), grade 2 (RR 0.64; 95% CI 0.33 to 1.23; *p* = 0.1814; I^2^ = 0%; Supporting Information S1: Figure S6), and grade 3 residual food (RR 1.54; 95% CI 0.59 to 3.97; *p* = 0.3748; I^2^ = 0%; Supporting Information S1: Figure S6).

The BIIB group was associated with a higher incidence of bile reflux when compared to the RY group (RR 3.10; 95% CI 1.75 to 5.50; *p* < 0.0001; *I* ^2^ = 74%; Figure 3b).

### 3.5 Other Outcomes

There were no significant differences between groups for time to first exhaust (MD 0.19 days; 95% CI −0.48 to 0.85; *p* = 0.7983; *I* ^2^ = 97%, Supporting Information S1: Figure S7), length of hospital stay (MD 0.06 days; 95% CI −0.23 to 0.34; *p* = 0.7026; *I* ^2^ = 80%, Supporting Information S1: Figure S8), and time to liquid diet (MD 0.18 days; 95% CI −0.86 to 0.51; *p* = 0.6152; *I* ^2^ = 91%; Supporting Information S1: Figure S9).

### 3.6 Sensitivity Analyses

Leave‐one‐out sensitivity analyses for the outcome of anastomotic time revealed consistent results after omitting each individual trial. The results for the sensitivity analyses are presented in the Supporting Information S1: Figure S10.

### 3.7 Risk of Bias Assessment

All RCTs were categorized as some concerns for overall risk of bias based on the RoB2 risk assessment. The main reason for this assessment were protocol problems. Six observational trials were categorized as high overall risk of bias and three as moderate risk of bias based on the ROBINS‐I risk assessment. The main reasons for this assessment were problems due to confounding and problems with outcomes measurement and reporting. Risk of bias assessment is presented in Supporting Information S1: Figures S11 and S12.

There was evidence of potential small study effects (publication bias) by visual appraisal for the outcome of anastomotic time, since studies with similar weights were asymmetrically distributed against their standard error (Supporting Information S1: Figure S13).

## 4 Discussion

In this systematic review and meta‐analysis of 10 studies, we compared BIIB versus conventional RY in adult patients who underwent laparoscopic distal gastrectomy. BIIB was associated with a lower anastomotic time, lower intraoperative blood loss, and lower operative time. In addition, there was an increased risk of alkaline bile reflux in BIIB when compared to RY. There were no significant differences between the groups for gastritis, residual food, leakage rate, number of retrieved lymph nodes, time to first exhaust, and length of hospital stay.

Billroth II alone is associated with bile reflux because the biliary limb delivers bile into the stomach [2]. This reflux can result in recurrence of gastric cancer, reflux esophagitis, symptoms of early satiety, Barret's esophagus and anastomotic stricture, which can result in gastric outlet or small bowel obstruction [2, 6, 23, 24]. BII with Braun anastomosis was conceived by Heinrich Braun to reduce the effects of alkaline reflux. In our analysis, BIIB was associated with an increased alkaline bile reflux risk when compared to RY. In BIIB, an anastomosis is created between two loops of small bowel (enteroenteric anastomosis), so bile can flow through the anastomosis rather than back into the gastric remnant [5]. As a result, there is still a chance for patients to experience alkaline reflux as it relies on a pressure gradient for bile juices to effectively flow through efferent limb [2, 23, 25]. RY however, completely diverts bile and pancreatic secretions away from the gastric remnant thus, anatomically preventing bile reflux [6, 25]. The biliary and pancreatic secretions then re‐enter the enteric system distally at the jejunojejunostomy [6]. Although Braun entero‐enterostomy was intended to mitigate the effects of bile reflux, diversion bile and pancreatic secretions remains pressure dependent; variations in distal intraluminal pressure may result in retrograde bile reflux into the gastric remnant [2, 25].

At present, there is no clearly defined or standardized management algorithm specific to bile reflux following BIIB reconstruction [26]. Management is therefore typically approached in a stepwise manner, beginning with medical therapy and progressing to surgical intervention in patients who remain symptomatic despite conservative treatment [27]. Medical therapies including hydrotalcite, prokinetic agents, sucralfate and ursodeoxycholic acid (UDCA) have been used in the management of alkaline bile reflux [26, 27, 28]. A recent meta‐analysis by Wu et al. demonstrated that UDCA was associated with improvement in bile reflux–related symptoms, with an 8‐week treatment course showing greater efficacy than a 4‐week course [29]. From a surgical perspective, Vogel et al. demonstrated significant reductions in both symptomatic and scintigraphically detected bile reflux following Braun enteroenterostomy [30]. However, unlike RY reconstruction, bile diversion with Braun enteroenterostomy is dependent on a pressure gradient and may be overcome in the setting of increased distal intraluminal pressure, which likely explains the persistence of bile reflux in some patients. In cases of severe, refractory bile reflux, RY reconstruction may be considered as a definitive option, while recognizing the associated risks of Roux stasis syndrome and internal hernia formation [25].

In BIIB there are two anastomoses: the gastrojejunostomy, distal to ligament of Treitz and a side to side jejunojejunostomy [2, 31]. Although there are two anastomosis in BIIB and two reconstructions in RY, the RY reconstructions involves multiple more steps and the anastomosis are more complex to form as it involves more mesenteric dissection which may compromise blood supply to the anastomosis and increase the risk of leakage [6]. Our results align with this finding, showing a lower operative and anastomosis time for patients undergoing BIIB when compared to RY, suggesting less technical complexity of BIIB.

RY requires more small bowel mobilization and mesenteric dissection [6, 13, 14, 18, 20, 32, 33]. A Roux limb and a biliopancreatic limb (BP) are created distally from Treitz ligament using a linear stapler to divide the jejunum [6]. The BP limb holds the bile and pancreatic juices, it is completely separated from the stomach. Distally, another anastomosis is created between the BP and Roux limb. With that, more mesenteric dissection is needed to mobilize and ensure tension free Roux limb. In BIIB, less mesenteric dissection is performed, thus reducing the risk of internal hernia, mesenteric bleeding, and devascularization of the small bowel, which is important in maintaining the integrity of the anastomoses [2, 34]. Our study showed that patients who underwent BIIB had less intraoperative blood loss, maybe due to less surgical complexity and less need for mesenteric dissection.

We found that, although no significant differences were observed for leakage rate between groups, BIIB presented with a lower anastomotic and operative time when compared to RY. Reduced operative time is especially beneficial in patients who have ASA > 3, as this reduced exposure time to anesthesia reduces the risk of hypoventilation, atelectasis, myocardial depression, and postoperative delirium [35, 36, 37, 38, 39]. Furthermore, in patients who are ASA > 3, it may be prudent to avoid further general anesthesia for complications such as internal hernias, or anastomotic leak [36, 37].

Zhong et al. performed a modified BIIB with a different reinforcement suture technique [17]. Instead of reinforcing the anastomotic site and residual end with 3–0 inverted sutures for hemostasis, as in a traditional BIIB, Zhong et al. used a 3–0 reverse cutting suture to strengthen the proximal jejunal remnant at the entrance of the afferent limb and the closed gastric remnant [17]. This technique elevates the jejunum at the gastrojejunal anastomosis, helping to stabilize its position and prevent retroperistalsis, thereby reducing the risk of obstruction. By elevating the jejunal limb and applying this reinforcement stitch, the authors also suggest a potential reduction in postoperative torsion, anastomotic obstruction, and operative time. However, our sensitivity analysis showed that, when excluding this paper, our results remained consistent in regards to complications and operative time.

Our analysis showed that BIIB and RY presented similar rates of complications such as anastomotic and intra‐abdominal bleeding and dumping syndrome. Multiple studies have shown a risk of internal hernia and Roux stasis syndrome in RY, with internal hernia risk increasing when patients lose weight [6, 40, 41]. However, with BIIB, there is reduced risk of internal hernia as there is less mesenteric dissection required [2]. Also, given the absence of a Roux limb, there is no risk of Roux stasis syndrome in BIIB, which is characterized by delayed gastric emptying due to lack of coordination of the duodenal pacemaker cells (interstitial cells of Cajal) [6, 42, 43]. Patients who develop Roux stasis syndrome suffer from nausea and vomiting, abdominal bloating, and epigastric discomfort [42, 43, 44, 45]. Therefore, given the similar safety profile, shorter operative time, and no significant difference in grade of gastritis or residual food, BIIB may be a suitable alternative to RY.

Decisions regarding the optimal reconstructive approach after distal gastrectomy (BIIB vs. RY) are multifactorial and therefore individualized. In addition to reflux‐related outcomes, operative considerations, including operative duration, technical complexity, surgeon preference, and anticipated blood loss may influence reconstruction selection. In this meta‐analysis, BIIB reconstruction was associated with shorter operative time and reduced intraoperative blood loss compared with RY reconstruction. In patients with significant comorbidities or high anesthetic risk (ASA class IV or greater), minimizing operative duration and procedural complexity may be particularly relevant. In this setting, BIIB reconstruction may be considered in carefully selected high‐risk patients, with acknowledgment of the potential for residual bile reflux relative to RY diversion.

This study has limitations. First, the included studies were mostly retrospective studies, which could increase the risk of confounding, risk of bias, and heterogeneity in our results. To address this, we performed leave‐one‐out sensitivity analysis that found consistent results. Second, the absence of individual patient‐level data prevented a more in depth analysis, limiting our possibility of exploring subgroup analysis. Third, many of the studies included were published from East Asia which may not accurately reflect the demographic of the world population, and limit the generalizability of our findings. Lastly, there were no patients with ASA > 4, which could also limit our findings into this population.

## Author Contributions

**Wendy Chang:** conceptualization, writing – original draft, formal analysis, writing – review and editing. **Lucas Monteiro Delgado:** methodology, validation, writing – review and editing, software, formal analysis, data curation. **Jessica Ng:** writing – review and editing, supervision. **Bryan Tran:** writing – review and editing.

## Funding

Funding for Open Access is provided by The University of Queensland.

## Conflicts of Interest

The authors declare no conflicts of interest.

## Supporting information


Supporting Information S1

Supporting Information S2

## Data Availability

The data that supports the findings of this study are available in the supplementary material of this article.
