# Supplementary material for: Billroth II With Braun Anastomosis Versus Roux‐En‐Y Reconstruction Following Distal Gastrectomy: A Systematic Review and Meta‐Analysis
Source: World J Surg. 2026 Feb 10;50(3):693–702. doi: 10.1002/wjs.70256 (PMC13006769; doi:10.1002/wjs.70256)

**SUPPLEMENTAL MATERIAL 1**

**Table of Contents**

**Supplementary Table 1 –** Search strategy.

**Supplementary Figure S1 -** Forest plot: ileus.

**Supplementary Figure S2 –** Forest plot: intraabdominal bleeding.

**Supplementary Figure S3 –** Forest plot: pneumonia.

**Supplementary Figure S4 –** Forest plot: grade 1,2,3, and 4 gastritis.

**Supplementary Figure S5 –** Forest plot: grade 1,2, and 3 residual food.

**Supplementary Figure S6 –** Forest plot: bile reflux

**Supplementary Figure S7 –** Forest plot: time to first exhaust.

**Supplementary Figure S8 –** Forest plot: length of hospital stay.

**Supplementary Figure S9 -** Forest plot: time to liquid diet.

**Supplementary Figure S10 -** Leave-one-out sensitivity analysis: anastomotic time

**Supplementary Figure S11 -** Risk of bias assessment (RoB2).

**Supplementary Figure S12 -** Risk of bias assessment (ROBINS-I).

**Supplementary Figure S13 –** Funnel plot: anastomotic time

**Supplementary Table 1 –** Search strategy.

(“gastric cancer” OR gastrectomy) AND (“Billroth-II with Braun” OR B-2-B)

**Supplementary Figure S1 -** Forest plot: ileus.


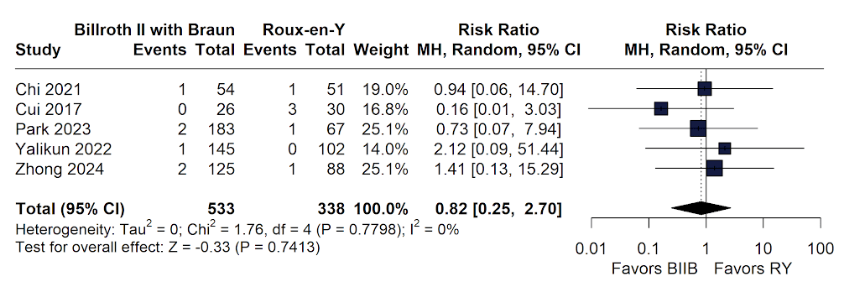


**Supplementary Figure S2 –** Forest plot: intraabdominal bleeding.


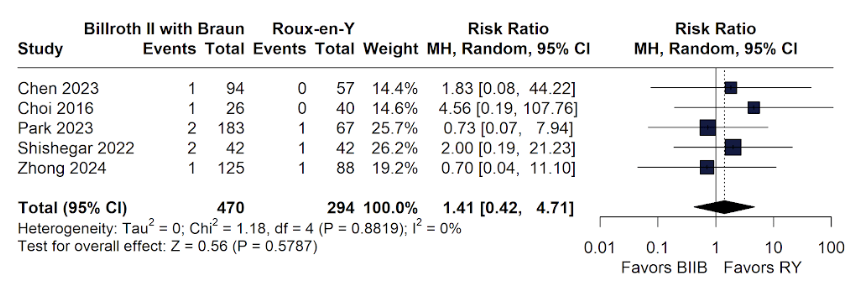


**Supplementary Figure S3 –** Forest plot: pneumonia.


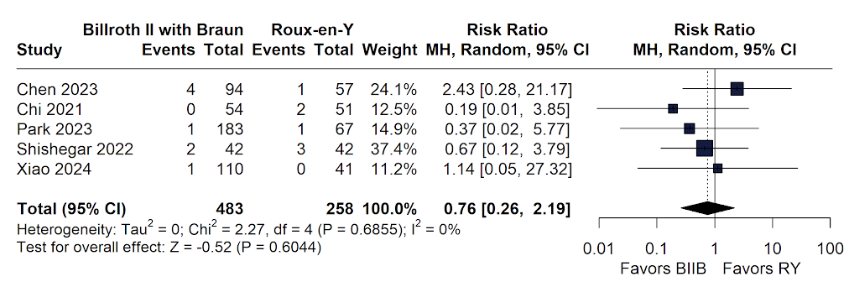


**Supplementary Figure S4 –** Forest plot: grade 1,2,3, and 4 gastritis.


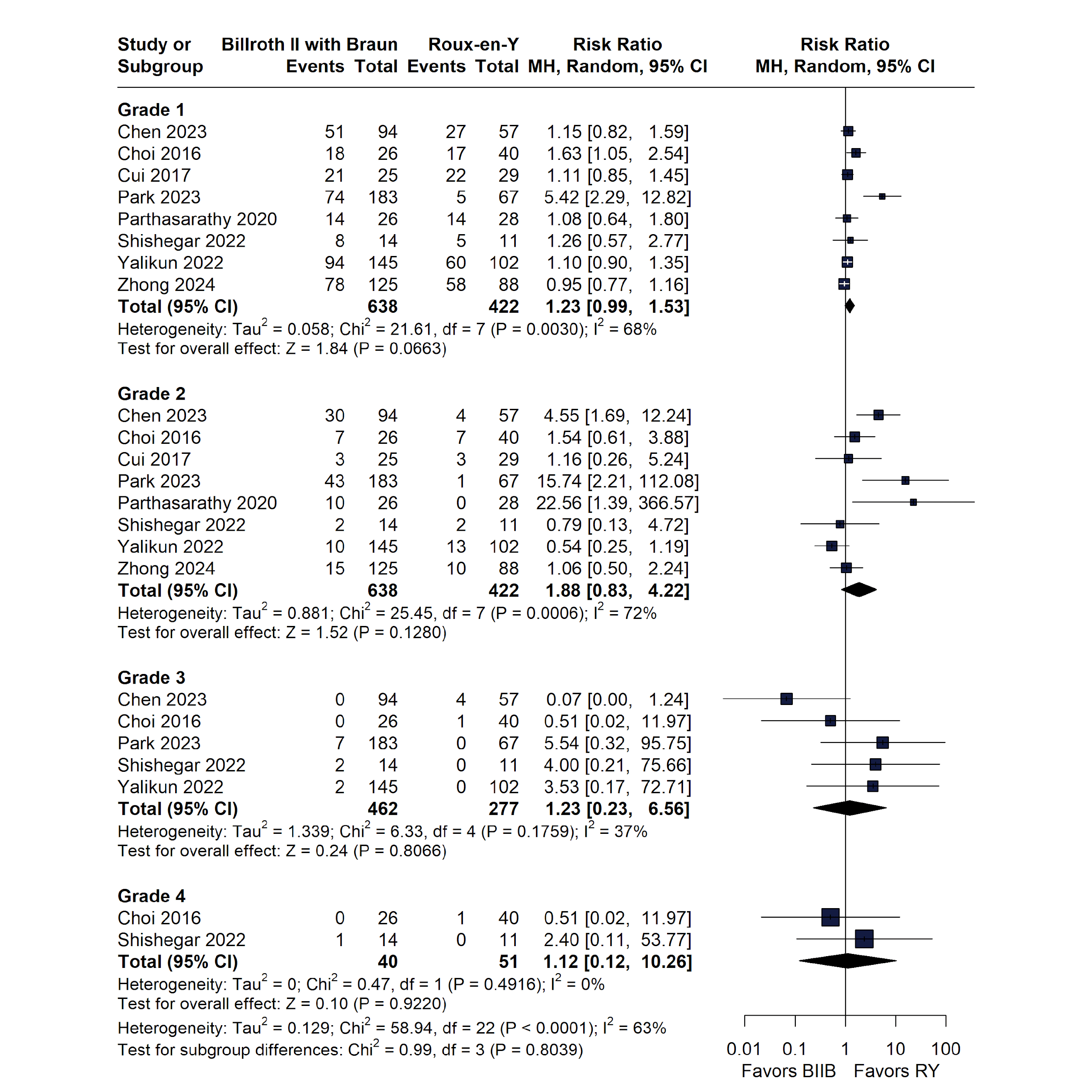


**Supplementary Figure S5 –** Forest plot: grade 1,2, and 3 residual food.

**
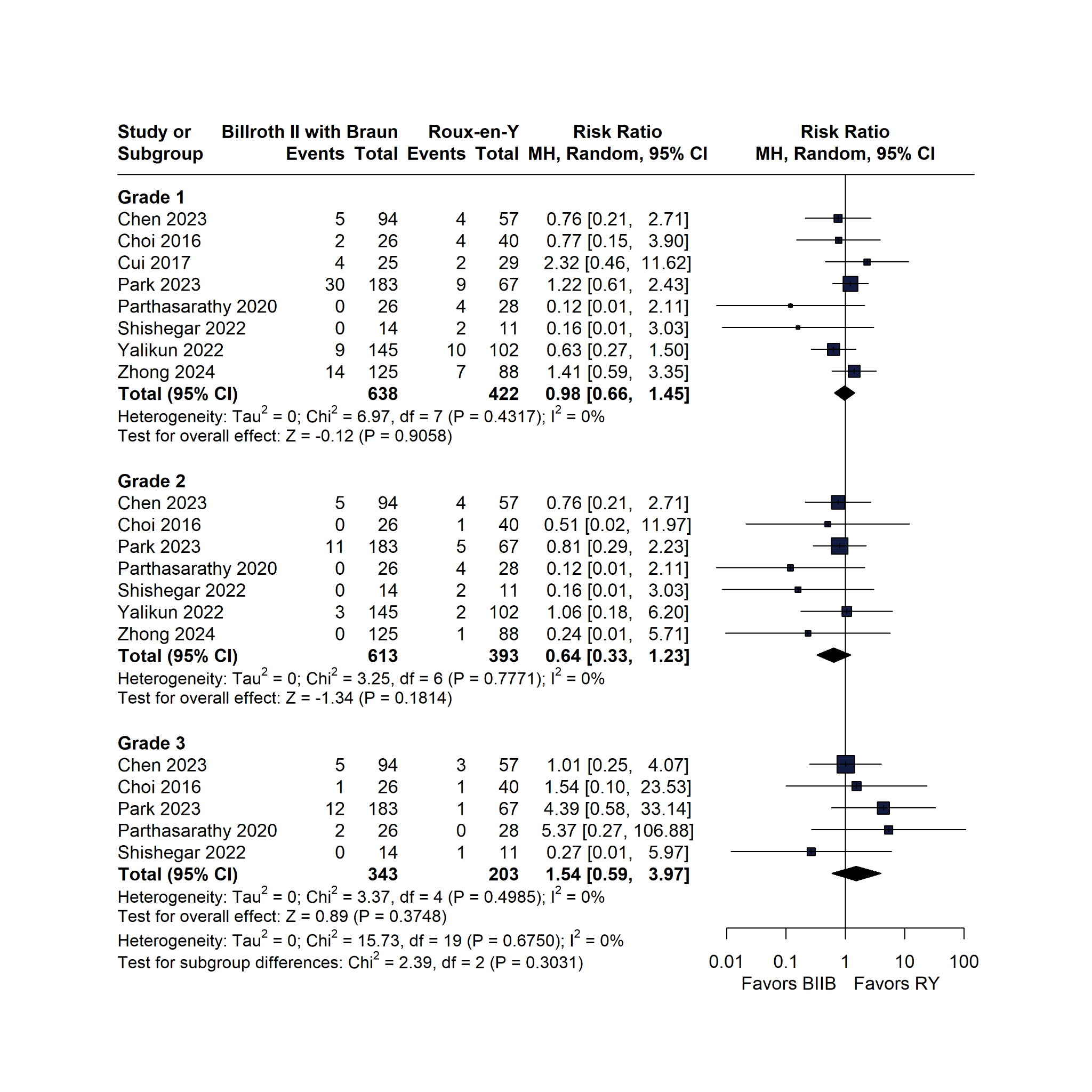
**

**Supplementary Figure S6 –** Forest plot: number of retrieved lymph nodes


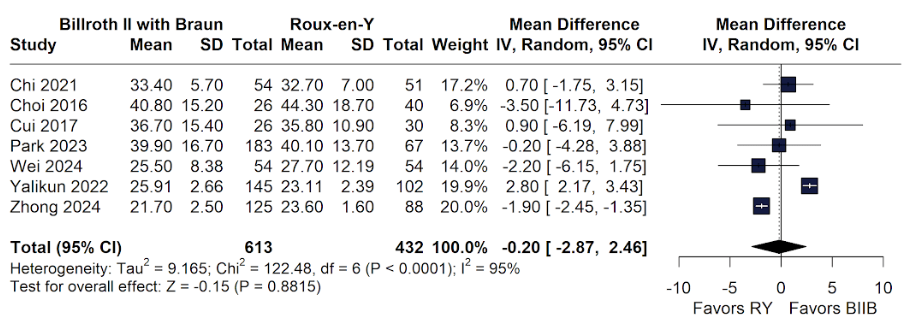


**Supplementary Figure S7 –** Forest plot: time to first exhaust.


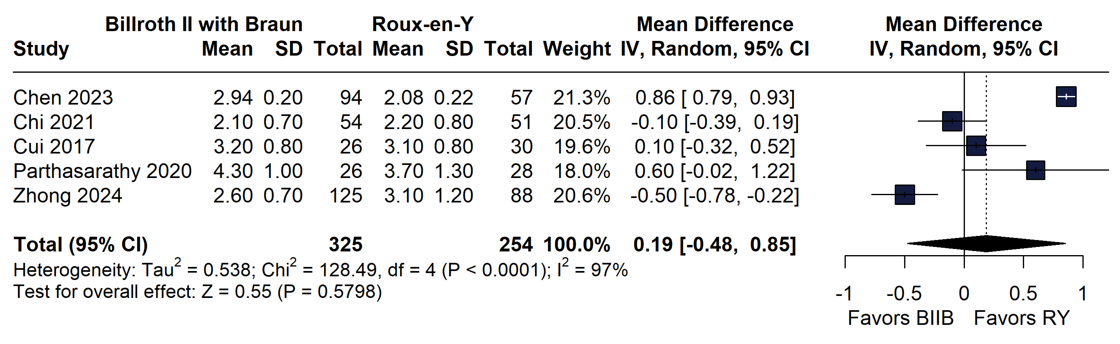


**Supplementary Figure S8 –** Forest plot: length of hospital stay.


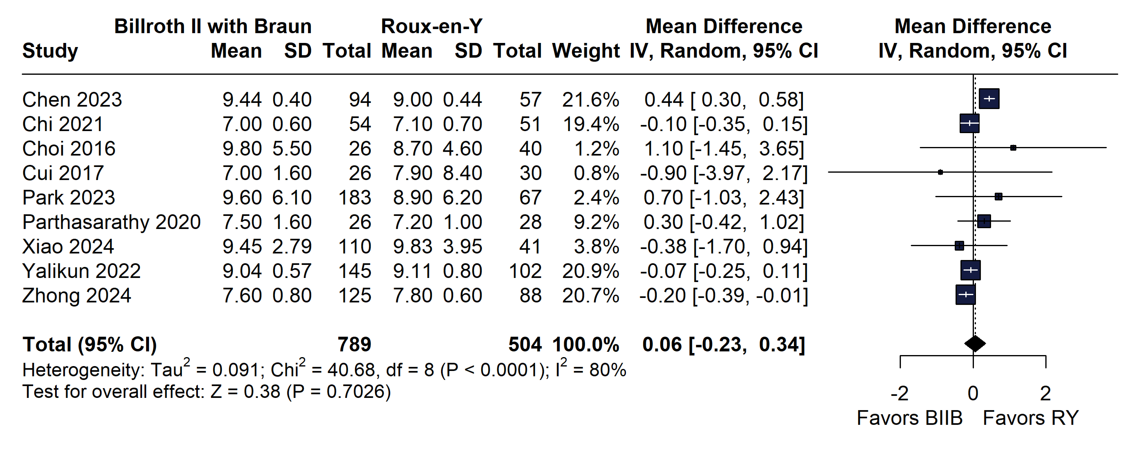


**Supplementary Figure S9 -** Forest plot: time to liquid diet.


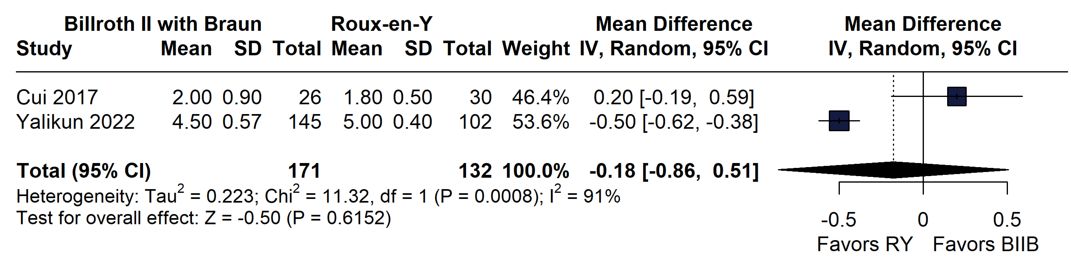


**Supplementary Figure S10 -** Leave-one-out sensitivity analysis: anastomotic time


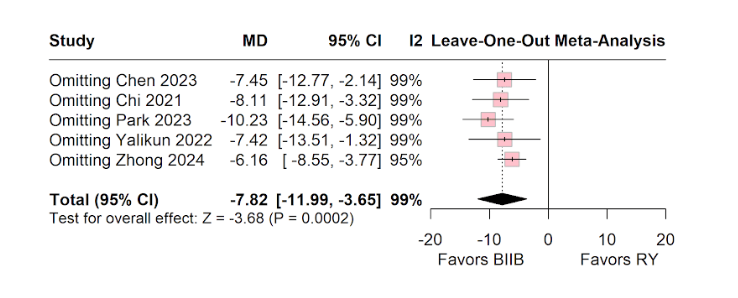


**Supplementary Figure S11 -** Risk of bias assessment (RoB2).


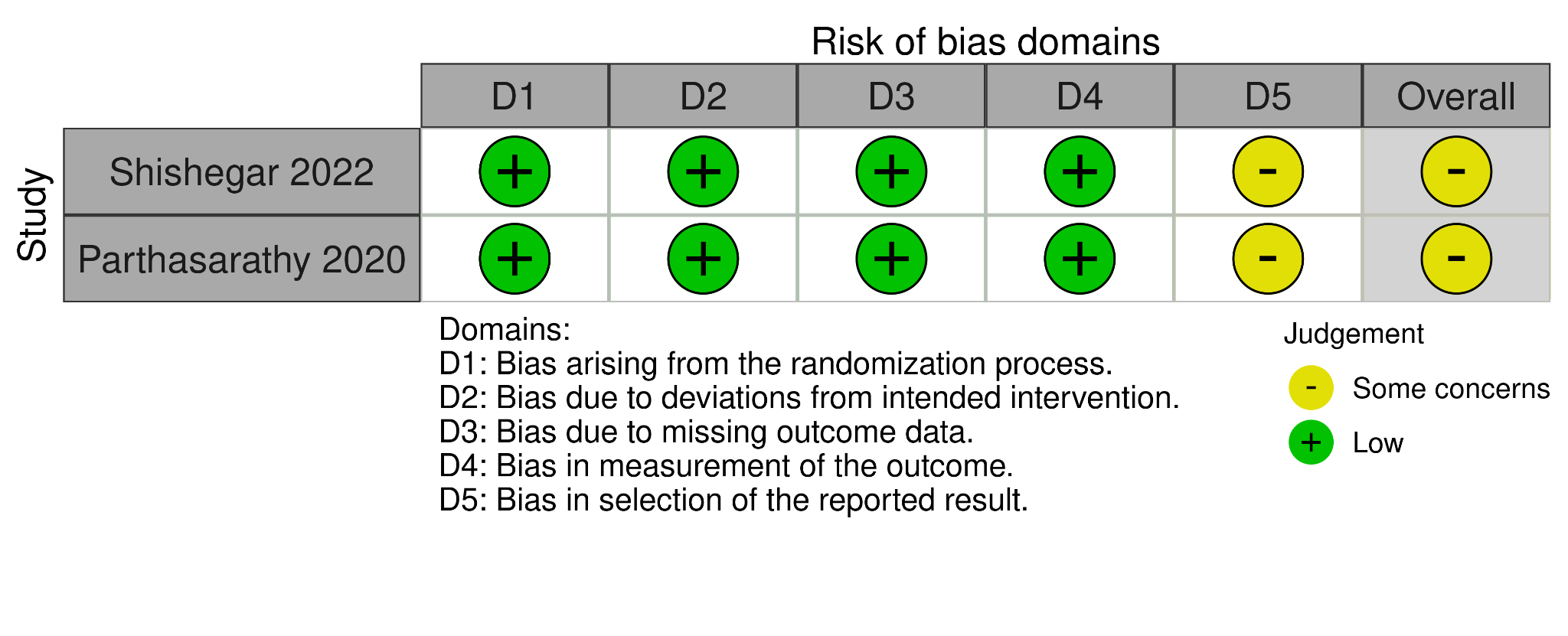


**Supplementary Figure S12 -** Risk of bias assessment (ROBINS-I).

**
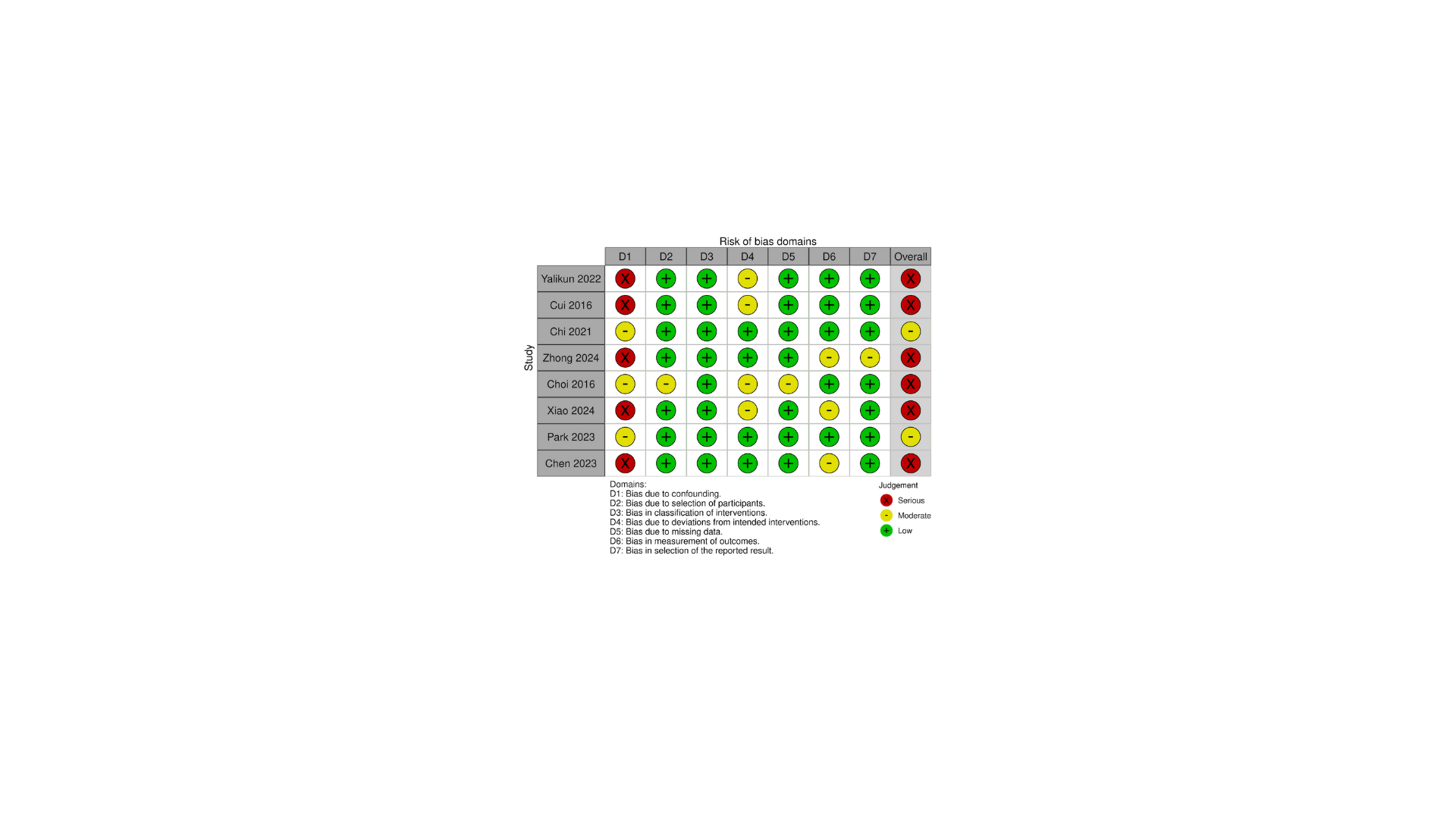
**

**Supplementary Figure S13 –** Funnel plot: anastomotic time


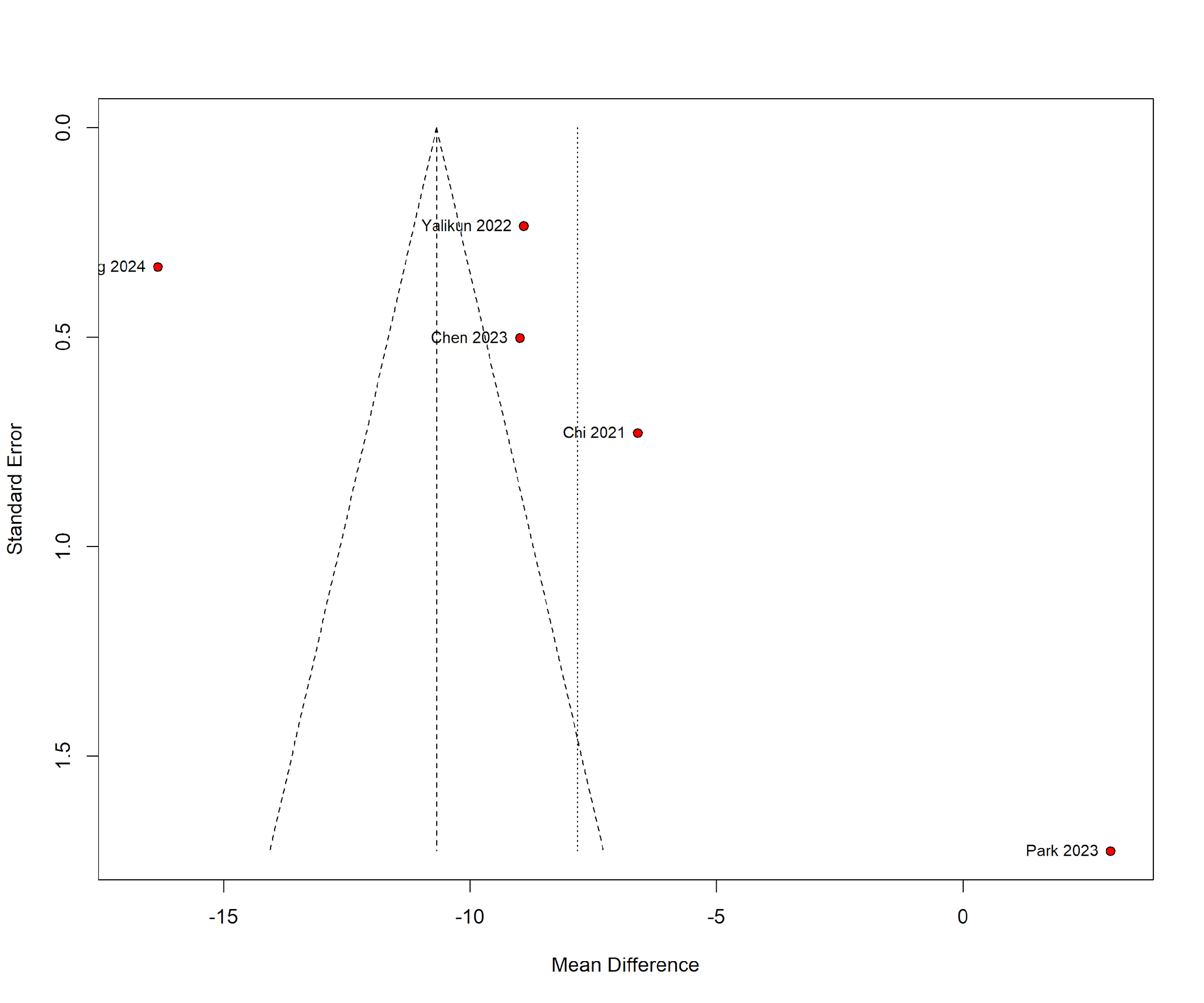

Supplement: Supplementary file 1 — Supporting Information S1 [file WJS-50-693-s002.docx]
